# SGLT‐2 Inhibitors for Ascites Management in Liver Cirrhosis: A Systematic Review and Meta‐Analysis of Available Evidence

**DOI:** 10.1155/ijh/7257876

**Published:** 2026-06-24

**Authors:** Abdallah Al Ghnaimat, Nour Aldin T. Ahmed, Omar Mahmood AL-AZZAWI, EzzElDien A. Ibrahim, Roaa Rayyan, Souheb Alkloub, Ekram Al Joufi, Ezz Eldein Breish, Sana Shaik, Noorhan Al-Kadhimi, Batool Al Masalha, Hossam Abdelbaqi, Dalia Atef Abouda

**Affiliations:** ^1^ Faculty of Medicine, Hashemite University, Zarqa, Jordan, hu.edu.jo; ^2^ Faculty of Medicine, Beni-Suef University, Beni-Suef, Egypt, bsu.edu.eg; ^3^ Faculty of Pharmacy, İstinye University, İstanbul, Turkey; ^4^ Faculty of Medicine, Cairo University, Cairo, Egypt, cu.edu.eg; ^5^ Faculty of Medicine, Zagazig University, Zagazig, Egypt, zu.edu.eg; ^6^ Faculty of Medicine, 21 September University for Medical and Applied Sciences, Sana′a, Yemen; ^7^ Faculty of Medicine, Kafr Elsheikh University, Kafr Elsheikh, Egypt; ^8^ Faculty of Medicine, Tbilisi State Medical University, Tbilisi, Georgia, tsmu.edu; ^9^ Faculty of Medicine, University of Jordan, Amman, Jordan, ju.edu.jo; ^10^ Faculty of Medicine, Tanta University, Tanta, Egypt, tanta.edu.eg; ^11^ Faculty of medicine, Alexandria university, Alexandria, Egypt, alexu.edu.eg

**Keywords:** ascites, dapagliflozin, diuretics, empagliflozin, liver cirrhosis, portal hypertension, SGLT-2 inhibitors, sodium-glucose transporter 2 inhibitors

## Abstract

**Goal:**

The goal is to systematically evaluate the efficacy and safety of SGLT‐2 inhibitors (SGLT2i) versus standard therapy for ascites management in liver cirrhosis.

**Background:**

SGLT2i promote natriuresis and osmotic diuresis, offering potential therapeutic benefits for fluid overload in cirrhosis, similar to their established role in heart failure. However, high‐quality comparative evidence remains limited.

**Study:**

A systematic search of PubMed, Scopus, Cochrane, Embase, and Web of Science was conducted through October 2025 following PRISMA 2020 guidelines. Eligible studies included randomized controlled trials and prospective comparative studies enrolling adults (≥ 18 years) with confirmed cirrhosis treated with dapagliflozin or empagliflozin, alone or alongside standard therapy, versus placebo or standard care for ≥ 2 weeks. The primary outcome was complete ascites resolution; secondary outcomes included mortality, body weight, eGFR, and serum creatinine and sodium levels.

**Results:**

Three studies (two RCTs and one prospective trial; *n* = 382) were included, comprising 241 patients receiving SGLT2i and 141 controls. SGLT2i significantly increased complete ascites resolution (OR: 2.39, 95% CI: 1.47–3.90; *p* < 0.001; *I* ^2^ = 15*%*) and reduced body weight (MD: −4.86 kg, 95% CI: −7.57 to −2.14; *p* = 0.0005; *I* ^2^ = 0*%*). No significant differences were observed in eGFR, serum creatinine, serum sodium, or mortality within the RCT subgroup (OR: 1.60, 95% CI: 0.53–4.87; *I* ^2^ = 0*%*); the overall three‐study pool (OR: 0.27, 95% CI: 0.13–0.56; *I* ^2^ = 87*%*) was driven by the nonrandomized trial (test for subgroup differences *p* = 0.001). The only placebo‐controlled RCT reported significantly higher rates of AKI (50% vs. 15%, *p* = 0.04) and infections (55% vs. 20%, *p* = 0.04) in the SGLT2i arm.

**Conclusions:**

SGLT2i improved ascites resolution and body weight without changes in routinely measured renal parameters, but unresolved AKI and infection signals and very low GRADE certainty for mortality preclude routine clinical use. SGLT2i should be considered only as individualized investigational therapy under close monitoring pending larger RCTs. PROSPERO (CRD420261303781).

## 1. Introduction

Liver cirrhosis represents the end stage of chronic liver disease. In 2017, cirrhosis caused more than 1.32 million deaths worldwide and affected over 10.6 million people with decompensated disease [1]. Ascites, the pathological accumulation of fluid within the peritoneal cavity, is the most common and serious complication of hepatic decompensation. It alters disease prognosis, as patients with cirrhosis experience significantly worsened survival after the first episode of decompensation [2]. Ascites significantly diminishes quality of life through abdominal discomfort, dyspnea, recurrent invasive procedures, and a high incidence of spontaneous bacterial peritonitis. Current first‐line management of cirrhotic ascites is dietary sodium restriction plus diuretic therapy, mainly mineralocorticoid receptor antagonists (typically spironolactone), with or without loop diuretics [3].

Fluid retention in cirrhosis primarily occurs due to peripheral arterial vasodilation driven by portal hypertension, leading to decreased effective circulating volume and subsequent activation of the renin‐angiotensin‐aldosterone system, sympathetic nervous system, and antidiuretic hormone secretion [4, 5]. Although this is a well‐established mechanism, conventional diuretic therapy has limitations. Refractory ascites occurs in approximately 5%–10% of patients and is defined as an inadequate response to maximum tolerated diuretic doses [6]. When medical therapy fails to manage ascites, patients require repeated paracentesis, an invasive procedure that carries major side effects such as postprocedural circulatory dysfunction, infection risk, and significant healthcare burden [7]. These limitations underscore the urgent need for therapeutic options that reduce ascites, improve disease outcomes, and carry fewer side effects.

Sodium‐glucose cotransporter‐2 (SGLT‐2) inhibitors are a class of drugs primarily used for glycemic control in patients with Type 2 diabetes mellitus (T2DM), with concurrent cardiovascular and renal benefits. By selectively inhibiting SGLT‐2 in the proximal renal tubule, these agents block glucose and sodium reabsorption, promoting glucosuria, natriuresis, and osmotic diuresis that reduce extracellular fluid volume and plasma volume [8]. Several trials regarding SGLT2 inhibitors (SGLT2is) with cardiovascular outcomes have established that SGLT‐2 inhibitors significantly reduce hospitalizations for heart failure and cardiovascular mortality in patients with Type 2 diabetes, independent of diabetes status and level of ejection fractions [9, 10]. SGLT‐2 inhibition improves tubuloglomerular feedback via increased sodium delivery to the macula densa, which suppresses the renin‐angiotensin‐aldosterone system [11]. Critically, these agents achieve volume reduction without directly activating the renin‐angiotensin‐aldosterone system.

The pathophysiological overlap between heart failure and cirrhotic ascites provides solid justification for testing SGLT‐2 inhibitors in patients with cirrhosis. Both conditions share the following fundamental characteristics: decreased effective blood volume, activation of the renin‐angiotensin‐aldosterone system, increased sympathetic nervous activity, and elevated antidiuretic hormone secretion. Together, these create a shared hemodynamic environment that responds to SGLT‐2 inhibition [12]. Heart failure and decompensated cirrhosis share not only overlapping neurohormonal activation pathways but also similar first‐line pharmacological management with mineralocorticoid receptor antagonists and loop diuretics [7, 9] [13]. The mechanistic parallels between these fluid‐retention states make it biologically sensible to consider these drugs as potential treatments for ascites in cirrhosis.

This systematic review and meta‐analysis aims to comprehensively evaluate the efficacy and safety of SGLT‐2 inhibitors in adult patients with liver cirrhosis and ascites. The primary objective was complete resolution of ascites. The secondary objectives were to determine the effects on disease progression markers, such as eGFR, serum sodium, and creatinine levels. Through this comprehensive evaluation, we aimed to address the current deficit of pooled evidence regarding SGLT‐2 inhibitor use in cirrhotic populations with fluid retention. Despite growing interest, no quantitative meta‐analysis has specifically focused on ascites outcomes in this population.

## 2. Methods

### 2.1. Study Design and Protocol Registration

We conducted this systematic review following PRISMA 2020 reporting standards [14] and Cochrane Handbook methodology [15]. The protocol was prospectively registered in PROSPERO (registration number: [CRD420261303781]). The review was conducted according to the registered protocol with no deviations.

### 2.2. Literature Search

We systematically searched five databases (PubMed, Scopus, Cochrane Library, Embase, and Web of Science) from their inception through October 2025. We developed a systematic search strategy to identify relevant studies that assessed the effects of SGLT2is on liver cirrhosis and ascites management. We searched for a combination of free‐text terms and controlled vocabulary related to SGLT2is and liver diseases. The following keywords were used: (“SGLT2” OR “sodium‐glucose cotransporter 2” OR “sodium glucose cotransporter 2” OR “dapagliflozin” OR “empagliflozin” OR “canagliflozin” OR “ipragliflozin”) AND (“cirrhosis” OR “liver cirrhosis” OR “hepatic cirrhosis” OR “end‐stage liver disease” OR “ascites” OR “fluid retention”). We supplemented this with manual screening of reference lists from included studies and relevant systematic reviews and searched trial registries (ClinicalTrials.gov, WHO International Clinical Trials Registry Platform) for ongoing or unpublished studies.

### 2.3. Eligibility Criteria

#### 2.3.1. Inclusion Criteria (PICO Framework)

Population: Adult patients (≥ 18 years) with clinically or histologically confirmed liver cirrhosis (Child‐Pugh Class A, B, or C). Intervention: SGLT2is, including ipragliflozin, dapagliflozin, empagliflozin, or canagliflozin, were administered alone or in combination with standard therapy for a minimum duration of 2 weeks. Comparator: Placebo or standard therapy without SGLT2is (e.g., insulin, standard medical therapy [SMT], standard of care, or other antidiabetic agents). Outcomes: Studies reporting outcomes related to ascites management (including ascites resolution, large‐volume paracentesis requirements, or clinical decompensation), metabolic parameters, renal function, or safety outcomes assessed at a minimum follow‐up of 2 weeks after baseline. Although RCTs were the primary study design of interest, one high‐quality prospective comparative trial was included given the limited randomized evidence in this emerging therapeutic area. This decision is transparently reported and acknowledged as the introduction of methodological heterogeneity.

#### 2.3.2. Exclusion Criteria

Studies were excluded if they met any of the following criteria: nonhuman or preclinical studies; observational studies (case‐control or cohort studies without prospective design), case reports, case series, reviews, meta‐analyses, conference abstracts, or editorials; studies that did not report relevant outcomes related to ascites management, metabolic parameters, renal function, or safety; studies involving patients with acute liver failure or severe renal impairment (eGFR <30 mL/min/1.73 m^2^); or studies that included patients receiving concurrent investigational hepatoprotective therapies.

### 2.4. Selection Process

Identified records were screened using Rayyan [16], a web‐based tool for systematic review management and screening of literature search results. We screened studies in two phases: The first phase was title/abstract screening for potential clinical studies. In the second phase, we retrieved the full‐text articles of the selected abstracts for further eligibility screening. Two independent reviewers performed literature searches and screenings at each phase. Any disagreements were resolved through discussion and, when necessary, through consultation with a third reviewer. We identified several ongoing registered clinical trials that evaluated SGLT2is in cirrhotic populations; however, none had published results at the time of our search. Three studies met the inclusion criteria and were included in the final analysis.

### 2.5. Data Extraction

We developed and pilot‐tested a standardized data extraction form on Google Sheets for randomly selected studies to ensure clarity and completeness before formal data extraction. Two reviewers independently extracted the data, and discrepancies were resolved by consensus or adjudication by a third reviewer. The relevant extracted data included study characteristics such as study ID, design, setting, population, intervention, control, and duration. Baseline characteristics included age, sex, weight, BMI, cirrhosis severity, ascites grade, diabetes status, laboratory values, and concomitant medications. Outcome data included primary outcomes (complete and partial ascites resolution), secondary outcomes (body weight, renal function parameters, electrolytes, and metabolic markers), and adverse events.

When outcome data were missing or unclear, we contacted corresponding authors via email. When multiple publications reported data from the same study population, we included the most recent or comprehensive report and supplemented it with additional details from earlier publications when necessary.

### 2.6. Study Risk of Bias Assessment

**Figure 1:**
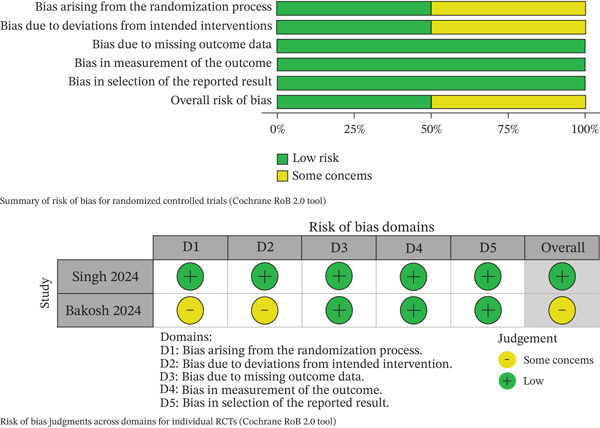
Summary of risk of bias and risk of bias judgments for randomized controlled trials assessed using the Cochrane Risk of Bias 2.0 tool.

We assessed the risk of bias in the included RCTs using the Cochrane Risk of Bias 2 (RoB 2) tool [17]. We evaluated studies as having low risk, some concerns, or high risk of bias according to five domains: bias arising from the randomization process, deviation from the intended intervention, missing outcome data, measurement of the outcome, and selection of the reported result. The Newcastle‐Ottawa Scale (NOS) was used for nonrandomized controlled studies, as shown in Figure 1 [18]. Two independent reviewers evaluated each study, with disagreements resolved by group discussion or consultation with a third author if needed.

### 2.7. Certainty of Evidence Assessment

The Grading of Recommendations, Assessment, Development, and Evaluation (GRADE) approach was used to assess the overall quality of evidence for each outcome of interest. This approach is based on five factors: the risk of bias, inconsistency, indirectness, imprecision, and publication bias. Confidence in the effect estimates is categorized as high, moderate, low, or very low according to the degree of certainty that the true effect lies close to the estimated effect. Evidence from RCTs was initially classified as high, but confidence may decrease based on the five factors listed above. Tables were constructed to present the certainty of the evidence and effect estimates for all primary outcomes, which serves as the summary of findings.

### 2.8. Statistical Analysis

A meta‐analysis was performed to synthesize data from RCTs evaluating the effectiveness and safety of SGLT2is in patients with liver cirrhosis and ascites. For quantitative synthesis, we pooled data from both randomized controlled trials (Singh et al. 2024 [19], Bakosh et al. 2024 [20]) and one prospective comparative trial (El‐Din et al. 2024 [21]) because of the limited number of available studies in this emerging therapeutic area.

#### 2.8.1. Software and Statistical Methods

All statistical analyses and forest plots were conducted using Review Manager (RevMan) Version 5.4. A fixed‐effects model using the inverse variance (IV) method was applied for all continuous outcomes, and the Mantel–Haenszel (M‐H) method was applied for dichotomous outcomes. Mean differences (MDs) with 95% confidence intervals (CIs) were calculated for continuous outcomes (body weight, serum creatinine level, estimated glomerular filtration rate [eGFR], and serum sodium level). Odds ratios (ORs) with 95% CI were calculated for dichotomous outcomes (ascites complete response and mortality). For mortality, a subgroup analysis stratified by study design (RCT vs. prospective study) was performed with a formal test for subgroup differences, given the methodological heterogeneity introduced by El‐Din et al. (nonrandomized, insulin comparator, 100% diabetic).

#### 2.8.2. Assessment of Heterogeneity

Interstudy heterogeneity was quantitatively evaluated using Cochran′s *Q* test (chi^2^ statistic) and *I* ^2^ statistic. We categorized heterogeneity as follows: *I* ^2^ = 0*%* indicating the absence of observed statistical heterogeneity, as observed in the body weight, eGFR, serum sodium, and RCT‐subgroup mortality analyses. *I* ^2^ = 25*%*–50*%* indicated low to moderate heterogeneity. An *I* ^2^ > 50*%* indicated substantial heterogeneity, as noted in the serum creatinine analysis (chi^2^ = 3.27, df = 1, *p* = 0.07; *I* ^2^ = 69*%*).

#### 2.8.3. Subgroup and Sensitivity Analyses

We determined the overall treatment effect using *Z*‐statistics, with a two‐tailed *p* value < 0.05 considered statistically significant. Prespecified subgroup analyses were performed based on follow‐up duration (3 months vs. 6 months) to assess the longitudinal consistency of clinical outcomes. Sensitivity analyses were planned to assess the robustness of the findings by (1) excluding studies at a high risk of bias and (2) comparing fixed‐effects and random‐effects models.

#### 2.8.4. Assessment of Publication Bias

Publication bias assessment using funnel plots and Egger′s regression test was planned when ≥ 10 studies were available for the meta‐analysis. Given the limited number of included studies, a formal statistical assessment of publication bias was not feasible.

## 3. Results

### 3.1. Literature Search

**Figure 2:**
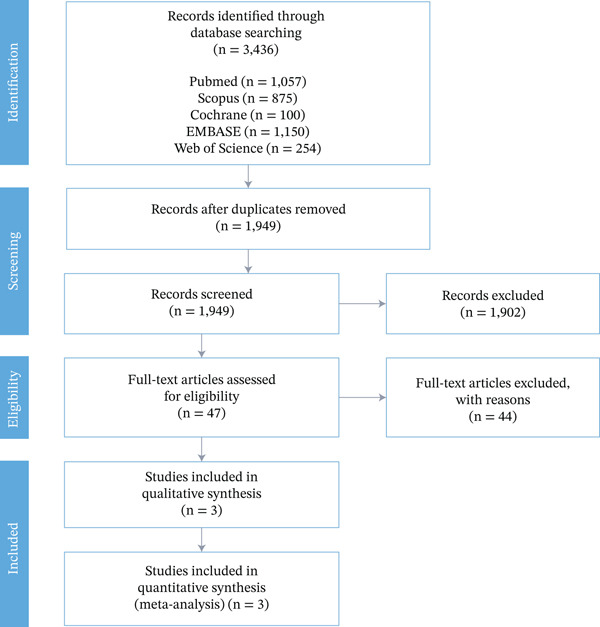
PRISMA 2020 flow diagram for the systematic review and meta‐analysis of SGLT‐2 inhibitors for ascites management in patients with liver cirrhosis.

A systematic search of the five databases yielded 3436 records (PubMed: 1057; Scopus: 875; Cochrane: 100; Embase: 1150; Web of Science: 254). After removing 1487 duplicates, 1949 records were screened by title and abstract, of which 1902 were excluded. The remaining 47 full‐text articles were assessed for eligibility; 44 were excluded with reasons. Three studies (two randomized controlled trials and one prospective comparative trial) met the inclusion criteria and were included in both the qualitative and quantitative synthesis [19–21], involving a total of 382 patients with liver cirrhosis. The pooled population consisted of 241 patients in the SGLT2i arm and 141 in the control arm, as illustrated in the PRISMA flow diagram (Figure 2).

### 3.2. Study Characteristics of Included Studies

The qualitative synthesis included three studies summarized in Table 1. Two randomized controlled trials (Singh et al. 2024 [19], Bakosh et al. 2024 [20]) and one prospective comparative trial (El‐Din et al. 2024 [21]). The studies were conducted across diverse geographical settings, including tertiary hepatology centers in India and Egypt. The specific therapeutic regimens varied among the trials, two studies evaluated dapagliflozin (Singh: 10 mg daily; El‐Din: 10 mg for Child A/B, 5 mg for Child C), and one evaluated empagliflozin (Bakosh: 10 mg daily). Treatment duration ranged from a minimum of 12 weeks (El‐Din) to a maximum of 6 months (Singh). All studies utilized a standard of care or placebo control, with background therapies including SMT or specific diuretic regimens.

**Table 1:** Characteristics of included studies.

Study	Design	Setting	Population	Intervention	Control	Duration	Key findings
Singh et al. 2024 [19]	Double‐blind RCT	Tertiary Hepatology Center, India	Adults with cirrhosis and recurrent Grade 3 ascites (*n* = 40)	Dapagliflozin 10 mg/day + SMT (*n* = 20)	Placebo + SMT (*n* = 20)	6 months	Improved ascites control and natriuresis (*p* < 0.001). No effect on CTP/MELD or survival (65% vs. 72%, *p* = 0.75). Higher AKI (50% vs. 15%, *p* = 0.04) and infections (55% vs. 20%, *p* = 0.04).
El‐Din et al. 2024 [21]	Prospective comparative trial	National Liver Institute, Menoufia University, Egypt	Adults with cirrhosis and T2DM (Child A–C) (*n* = 300)	Dapagliflozin (10 mg for Child A/B; 5 mg for Child C) + insulin (*n* = 200)	Insulin alone (*n* = 100)	12 weeks	Improved ascites, reduced diuretic dose, and lower Child–Pugh scores (*p* = 0.0001). Significant weight and BMI reduction. Lower hypoglycemia, encephalopathy, and variceal bleeding vs. insulin.
Bakosh et al. 2024 [20]	Randomized, investigator/assessor‐blinded controlled trial	Hepatology Unit, Alexandria University, Egypt	Adults with cirrhosis and refractory Grade 3 ascites (Child B/C) (*n* = 42)	Empagliflozin 10 mg/day + SoC (*n* = 21)	SoC alone (*n* = 21)	3 months	Eliminated LVP need in 57.1% (vs. 0% SoC, −). Complete ascites resolution in 24%. AKI more common in SoC group. Mild hyponatremia and muscle cramps with empagliflozin.

*Note:* This table summarizes the design, population, interventions, and key findings of all studies included in the systematic review.

Abbreviations: AKI, acute kidney injury; CTP, Child–Turcotte–Pugh; LVP, large‐volume paracentesis; MELD, model for end‐stage liver disease; RCT, randomized controlled trial; SMT, standard medical therapy; SoC, standard of care; T2DM, Type 2 diabetes mellitus.

### 3.3. Baseline Characteristics of the Included Studies

Baseline demographic and clinical characteristics of the pooled population are summarized in Table 2. The mean age of participants ranged from 49.7 to 65.7 years, with a male predominance observed in the Singh cohort, whereas the El‐Din and Bakosh cohorts showed a more balanced or female‐predominant sex distribution.

**Table 2:** Baseline characteristics of included studies.

Study	Age (years)	Male *n* (%)	Weight (kg)	BMI (kg/m^2^)	Child–Pugh class	Child–Pugh score	MELD score	MELD‐Na score	Ascites grade	T2DM n (%)	HbA1c (%)	Hb (g/dL)	Creat (mg/dL)	eGFR (mL/min)	Na (mEq/L)	Diuretics n (%)	Prior LVPs
Singh 2024 [19](*n* = 40)	51.4 ± 8.5	35 (87.5)	NR	NR	NR	10.5 ± 2.6	22.0 ± 4.5	22.0 ± NR	Grade 3 recurrent	3 (7.5)	5.0 ± 0.6	9.1 ± NR	1.5 ± 0.5	NR	NR	40 (100)	4.0 ± 1.1
El‐Din 2024 [21](*n* = 300)	56.9 ± 8.2	140 (46.7)	93.2 ± 18.2	32.6 ± 5.7	A: 80, B: 113, C: 107	8.1 ± NR	11.8 ± 5.4	16.0 ± 6.8	Mixed	300 (100)	9.9 ± 1.9	NR	0.93 ± 0.40	162.6 ± 86.3	132.5 ± 5.3	250 (83.3)	NR
Bakosh 2024 [20](*n* = 42)	65.4 ± 5.0	20 (47.6)	91.8 ± 11.7	NR	B: 23, C: 19	9.0 ± 1.0	NR	16.0 ± 5.7	Grade 3 refractory	20 (47.6)	NR	10.1 ± 1.0	1.2 ± 0.3	59.9 ± 18.2	135.0 ± 6.1	42 (100)	NR

*Note:* The table presented the baseline demographic, clinical, and laboratory characteristics of study populations. Values are presented as mean ± SD or *n* (%) unless otherwise specified. Data pooled across intervention and control groups where applicable.

Abbreviations: Bili, bilirubin; BMI, body mass index; Creat, creatinine; eGFR, estimated glomerular filtration rate; Hb, hemoglobin; HbA1c, glycated hemoglobin; LVPs, large‐volume paracenteses; MELD, model for end‐stage liver disease; Na, sodium; NR, not reported; NSBBs, nonselective beta blockers; T2DM, Type 2 diabetes mellitus.

### 3.4. Quality Assessment

Risk of bias was assessed using the Cochrane RoB 2 tool for the two RCTs and the NOS for the prospective comparative trial. Singh et al. 2024 was rated as having some concerns overall, primarily arising from the randomization domain due to limited reporting of allocation concealment details. Bakosh et al. 2024 was rated as low risk across all five domains, with adequate sequence generation, blinding of outcome assessors, and complete outcome data reporting. Across both RCTs, no domains were judged as high risk of bias. El‐Din et al. 2024, assessed using the NOS, achieved a score of 7 out of 9 stars, reflecting satisfactory selection of exposed and nonexposed cohorts, adequate comparability based on key confounders, and complete ascertainment of outcomes, with minor limitations related to the nonrandomized design and potential for residual confounding. The overall risk of bias profile across the included studies is illustrated in Figure 1.

The severity of liver disease and metabolic comorbidities varied significantly across the included trials.

#### 3.4.1. Hepatic Status

Singh et al. (2024) and Bakosh et al. (2024) specifically enrolled patients with advanced decompensation, characterized by Grade 3 or refractory ascites and higher MELD scores (ranging from approximately 17 to 22), whereas El‐Din et al. (2024) included a broader spectrum of cirrhosis severity (Child–Pugh A, B, and C) with mixed ascites grades.

#### 3.4.2. Comorbidities

The prevalence of T2DM was 100% in the El‐Din trial, which focused on patients of diabetes and cirrhosis. Conversely, T2DM prevalence was lower in the ascites‐focused trials (5%–10% in Singh et al. 2024 and approximately 43%–52% in Bakosh et al. 2024).

#### 3.4.3. Renal Function

Baseline serum creatinine levels were generally within the range of 0.87–1.5 mg/dL.

#### 3.4.4. Concomitant Medication

Diuretic use was reported to be 100% in the Singh and Bakosh trials, which is consistent with the recruitment of patients with significant fluid overload.

### 3.5. Narrative Synthesis

El‐Din et al. 2024 [21] (*n* = 300) conducted a prospective comparative trial evaluating dapagliflozin versus insulin in cirrhotic patients with Type 2 diabetes. The study demonstrated that dapagliflozin treatment resulted in significant improvements in ascites status, with a markedly reduced need for diuretic dose escalation compared with insulin therapy. Additionally, dapagliflozin was associated with significant reductions in body weight (MD: −4.1 kg), BMI, and fasting blood glucose. The incidence of adverse events, including hypoglycemia, hepatic encephalopathy, variceal bleeding, and urinary tract infection, was significantly lower in the dapagliflozin group than in the insulin group. This study was included in the quantitative meta‐analysis because of its prospective comparative design and the limited number of available RCTs in this emerging therapeutic area, with the acknowledgment that this introduces some methodological heterogeneity.

### 3.6. Efficacy Outcomes: Ascites Resolution and Fluid Mobilization

**Figure 3:**
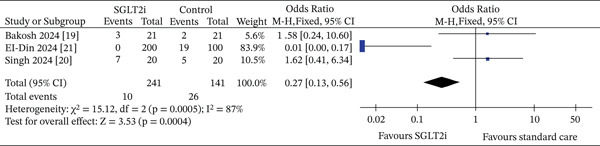
Forest plot showing the effect of SGLT‐2 inhibitors versus standard care on complete ascites resolution in patients with liver cirrhosis and ascites.

The primary clinical endpoint, complete resolution of ascites, was analyzed in three studies (Singh et al. 2024 [19], El‐Din et al. 2024 [21], Bakosh et al. 2024 [20]; total *n* = 382), as shown in Figure 3. The pooled analysis showed an OR of 2.39 (95% CI: 1.47–3.90, *p* < 0.001), with low heterogeneity (*I* ^2^ = 15*%*), indicating significantly higher rates of complete ascites response in the SGLT2i group compared to standard care. Subgroup analysis by follow‐up duration demonstrated consistent efficacy; at 3 months, 44.8% of patients in the SGLT2i group achieved complete response versus 24.8% in the control group (OR: 2.28, 95% CI: 1.39–3.75, *p* = 0.001).

SGLT2i treatment also resulted in a significantly greater reduction in body weight, serving as a surrogate marker for effective fluid mobilization. The MD between groups was −4.86 kg (95% CI: −7.57 to −2.14, *p* = 0.0005) favoring the intervention arm.

### 3.7. Renal Function and Electrolyte Safety

Renal parameters showed a trend toward renoprotection. Serum creatinine levels remained stable, with no significant difference between the groups (MD: −0.05 mg/dL, 95% CI: −0.14 to 0.04, *p* = 0.30), as shown in Figure S1. Patients receiving SGLT2i therapy showed a nonsignificant improvement in the estimated Glomerular Filtration Rate (eGFR) compared to controls (MD: 10.46 mL/min/1.73m^2^, 95% CI: −0.70 to 21.62, *p* = 0.07), as shown in Figure S2. Furthermore, SGLT2i administration did not significantly alter serum sodium levels (MD: −0.54 mmol/L, 95% CI: −1.73 to 0.66, *p* = 0.38), suggesting no increased risk of treatment‐induced hyponatremia.

### 3.8. Mortality

**Figure 4:**
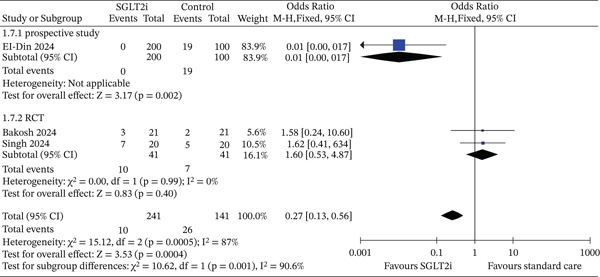
Forest plot showing the effect of SGLT‐2 inhibitors versus standard care on mortality in patients with liver cirrhosis and ascites, stratified by study design (RCT subgroup vs. prospective study). Test for subgroup differences: chi^2^ = 10.62, *p* = 0.001, *I* ^2^ = 90.6*%*.

Mortality data were available from all three studies (*n* = 382), analyzed as a subgroup analysis by study design (Figure 4). Within the RCT subgroup, pooled mortality did not differ between groups (Singh [19] 7/20 vs. 5/20; Bakosh [20] 3/21 vs. 2/21; OR: 1.60, 95% CI: 0.53–4.87; *p* = 0.40; *I* ^2^ = 0*%*). The prospective study subgroup (El‐Din [21]; 0/200 vs. 19/100) reported a markedly different effect (OR: 0.01, 95% CI: 0.00–0.17). The test for subgroup differences was highly significant (chi^2^ = 10.62, *p* = 0.001; *I* ^2^ = 90.6*%*). The overall three‐study pool (OR: 0.27, 95% CI: 0.13–0.56; *I* ^2^ = 87*%*) is driven by El‐Din et al. (83.9% weight) and reflects between‐design heterogeneity rather than a class‐wide effect; the RCT subgroup is the primary mortality estimate.

#### 3.8.1. Statistical Analysis Rationale

Data synthesis was performed using the RevMan software. We employed the fixed‐effect model (M‐H method for dichotomous data and IV for continuous data) for the meta‐analysis of all outcomes. This model selection was substantiated by the low level of statistical heterogeneity observed among the included studies:

#### 3.8.2. Negligible Heterogeneity

For the outcomes of body weight, serum sodium, eGFR, and RCT‐subgroup mortality, the *I* ^2^ statistic was exactly 0% (*p* > 0.05 for chi‐squared test), indicating high consistency in effect sizes across the trials.

#### 3.8.3. Low Heterogeneity in Primary Outcome

The primary efficacy endpoint, ascites complete response, demonstrated low heterogeneity (*I* ^2^ = 15*%*), further justifying the use of a fixed‐effect approach as the variation in study results is likely attributable to sampling error rather than methodological diversity.

## 4. Discussion

This systematic review and meta‐analysis showed that SGLT2is improve ascites resolution in patients with liver cirrhosis, with 2.4‐fold higher odds of complete ascites resolution compared to standard therapy and a weight reduction of nearly 5 kg. Renal safety was favorable, with trends toward improved eGFR and stable serum creatinine and sodium levels.

Previous systematic reviews examined SGLT2is in cirrhosis broadly, focusing on disease progression, hemodynamics, and metabolic parameters [22, 23]. Dhoop et al. [22] conducted a systematic review of 16 studies and performed a narrative synthesis without a meta‐analysis due to heterogeneity. They found consistent ascites reduction across all decompensated cirrhosis studies, with weight loss ranging from 3.4 to 7.5 kg, but noted safety concerns including AKI risk in two of nine studies and increased infections in two of five studies. Our findings agree with efficacy: the pooled analysis confirmed significant ascites resolution (OR: 2.39, 95% CI: 1.47–3.90, *p* < 0.001) and weight reduction (MD: −4.86 kg, *p* = 0.0005). However, our pooled data showed no statistically significant increase in AKI, suggesting a more favorable renal profile than individual case reports. The key difference is that our quantitative meta‐analysis of RCTs provided precise effect estimates, whereas Dhoop et al. relied on narrative synthesis across heterogeneous study designs. Our analysis was specifically designed to address ascites as a manifestation of portal hypertension and advanced decompensation. Although some included trials overlap with earlier reviews, our study differs in its predefined objectives, eligibility criteria, and outcome hierarchy, and therefore provides complementary rather than redundant evidence. Separately, Mantovani et al. [23] pooled eight observational cohort studies with over 626,000 patients with Type 2 diabetes and found that SGLT2i use was linked to a lower risk of major adverse liver outcomes (HR: 0.83, 95% CI: 0.72–0.95) and liver‐related deaths (HR: 0.64, 95% CI: 0.50–0.82). Several other large observational studies have reported reduced risks of hepatic decompensation, hepatorenal syndrome, and mortality among patients with cirrhosis on SGLT2is [24–26].

The studies included in this review all favored SGLT2is for ascites, but differed in population severity, comparators, and safety findings. Singh et al. [19] conducted the only double‐blind, placebo‐controlled RCT that enrolled patients with recurrent Grade 3 ascites, and reported better ascites control and higher natriuresis with dapagliflozin. Simultaneously, this trial reported a higher incidence of AKI (50% vs. 15%) and infections (55% vs. 20%) in the dapagliflozin arm. Most AKI episodes occurred alongside sepsis; therefore, it is difficult to know how much of that risk is due to the drug itself. Bakosh et al. [20] studied empagliflozin in refractory ascites and found the opposite pattern for AKI—with lower rates in the empagliflozin group (33.3% vs. 57.1% with standard of care alone). This discordance may stem from differences in the drugs used, disease severity, comparator (placebo vs. active standard of care), or chance variation in small trials. El‐Din et al. [21] enrolled the largest cohort (*n* = 300) and showed clear weight reduction and lower diuretic requirements with dapagliflozin across Child–Pugh Class A through C patients. However, this was a prospective comparative trial with insulin as comparator rather than a placebo‐controlled RCT, and this design difference should be considered when interpreting the effect sizes.

The pooled analysis included patients with Child–Pugh A cirrhosis with diabetes (El‐Din et al. 2024 [21]) and Child–Pugh B/C patients with refractory Grade 3 ascites (Singh et al. 2024 [19], Bakosh et al. 2024 [20]). Despite these clinical differences, the statistical heterogeneity was low for most outcomes. Ascites resolution showed an *I* ^2^ of 15%, body weight *I* ^2^ = 0*%*, serum sodium *I* ^2^ = 0*%*, eGFR *I* ^2^ = 0*%*, and RCT‐subgroup mortality *I* ^2^ = 0*%*. Notable heterogeneity was observed in serum creatinine (*I* ^2^ = 69*%*) and in the overall three‐study mortality pool (*I* ^2^ = 87*%*, reflecting between‐design heterogeneity), which likely reflects the divergent renal responses in Bakosh et al. (creatinine improved with empagliflozin) versus El‐Din et al. (similar creatinine changes in both arms). These differences may be related to contrasting comparator arms and baseline renal profiles.

The weight loss observed provides objective evidence of fluid mobilization and may reduce the need for large‐volume paracentesis, which affects approximately 5%–10% of patients despite maximal diuretic therapy [27]. For patients with refractory ascites, TIPS has been shown to lower ascites recurrence and reduce the risk of hepatorenal syndrome, but it does not improve survival and is associated with more frequent severe hepatic encephalopathy and higher costs compared with repeated paracentesis plus albumin [28]. SGLT2is may offer a less invasive alternative in selected patients, though head‐to‐head comparisons are lacking. The mechanism of action of SGLT2is in cirrhotic ascites differs from that of conventional diuretics. Standard management includes aldosterone antagonists and loop diuretics that act on the distal nephron and collecting duct. SGLT2is target the proximal tubule, where they promote natriuresis and osmotic diuresis through glycosuria without directly stimulating the RAAS [12, 21] [27]. Because they act on different nephron segments, they may still work in patients who do not respond to distal‐acting diuretics.

On the safety side, our meta‐analysis found no significant change in serum creatinine (MD: −0.05 mg/dL, 95% CI: −0.14 to 0.04, *p* = 0.30) or sodium (MD: −0.54 mmol/L, 95% CI: −1.73 to 0.66, *p* = 0.38), and a trend toward higher eGFR (MD 10.46 mL/min/1.73m^2^, 95% CI: −0.70 to 21.62, *p* = 0.07). These results are in line with the renoprotective effects seen in cardiovascular and diabetic populations [29]. A 48‐month study in patients with CTP‐B cirrhosis showed that SGLT2i therapy improved GFR compared to insulin, with patients moving from CKD Stage 3a to Stage 2 [30]. Still, the safety data are not entirely reassuring. The Singh et al. [19] trial found more AKI with dapagliflozin, and the review by Dhoop et al. [22] also reported hemodynamic instability and AKI in two of nine studies . Importantly, the null pooled creatinine/eGFR estimates do not capture AKI or infection (binary clinical events), and the significantly elevated rates of AKI (50% vs. 15%, *p* = 0.04) and infection (55% vs. 20%, *p* = 0.04) in the only placebo‐controlled RCT cannot be ruled out as class effects; close monitoring of renal function, volume status, and infection should be considered mandatory. This probably comes down to disease severity: patients with recurrent ascites who need frequent paracentesis are hemodynamically fragile, and any diuretic agent carries a risk in that setting.

Our mortality subgroup analysis showed no significant difference within the RCT subgroup (OR: 1.60, 95% CI: 0.53–4.87, *p* = 0.40, *I* ^2^ = 0*%*), whereas the single nonrandomized trial drove the overall pool (test for subgroup differences *p* = 0.001). The GRADE certainty was very low because the CI was wide enough to include both meaningful benefit and meaningful harm. The gap between these neutral short‐term findings in small RCTs and the favorable mortality signals from large observational cohorts likely reflects differences in follow‐up duration, patient selection, or the time needed for the disease‐modifying effects to become apparent.

This meta‐analysis is the first quantitative synthesis to specifically target ascites outcomes with SGLT2is in patients with cirrhosis. The review was prospectively registered on PROSPERO and followed the PRISMA 2020 guidelines. Screening, data extraction, and risk of bias assessment were performed independently by two reviewers, using the Cochrane RoB 2.0 tool for RCTs and NOS for nonrandomized studies. Pooling all available prospective evidence improved precision over individual trials, and the low heterogeneity across most outcomes (*I* ^2^ = 0*%*–15*%*) supported the consistency of the treatment effect. We also performed sensitivity analyses and GRADE assessment to evaluate the certainty of evidence.

However, there are important limitations to consider. The evidence base is still small, only two RCTs and one prospective comparative study were included, with a combined sample of 382 patients. This contributed to imprecision, as reflected in the GRADE ratings, moderate for ascites resolution and body weight, low for serum sodium, and very low for creatinine, eGFR, and mortality. The studies used different SGLT2is (dapagliflozin and empagliflozin) at different doses and with different comparators (placebo, standard of care, insulin); therefore, we cannot determine which drug or dose is best. The way ascites was assessed also varied, some trials used ultrasound grading, whereas others relied on clinical evaluation or the need for paracentesis. The follow‐up duration was short (3–6 months).

For carefully selected patients with refractory or diuretic‐resistant ascites, particularly those with concurrent diabetes and preserved renal function, our findings suggest that SGLT2is are a reasonable treatment option to consider within a multidisciplinary approach. Close monitoring of renal function, electrolyte levels, and infection risk remains essential, particularly in patients with advanced decompensation. There are several ongoing trials examining SGLT2is in patients with cirrhosis, which may expand the available evidence in coming years. Future studies should be larger and powered to assess quality of life, paracentesis frequency, hospitalizations, transplant‐free survival, and overall mortality. Stratifying by cirrhosis severity and diabetes status would help identify who benefits the most and who may face greater risk. Comparisons between different SGLT2is and dose‐finding studies are needed to guide prescriptions. Extending this research to non‐diabetic cirrhotic patients is also relevant, given that SGLT2is have already been approved for non‐diabetic heart failure. Long‐term safety data from real‐world cirrhotic cohorts, particularly regarding AKI, infections, and hemodynamic stability in advanced disease, remain a pressing gap.

## 5. Conclusions

In conclusion, this meta‐analysis provides the first quantitative synthesis showing that SGLT2is improve ascites resolution, reduce body weight, and appear safe from a renal standpoint. Given the limited evidence base, unresolved AKI and infection signals, and low to very low GRADE certainty for most outcomes other than ascites resolution, SGLT2is should be considered only as individualized investigational therapy under close monitoring; routine clinical use awaits validation by adequately powered RCTs.

**Nomenclature**
AKI: acute kidney injury
BMI: body mass index
CI: confidence interval
CKD: chronic kidney disease
CTP: Child–Turcotte–Pugh
eGFR: estimated glomerular filtration rate
GRADE: Grading of Recommendations, Assessment, Development, and Evaluation
HR: hazard ratio
MD: mean difference
MELD: model for end‐stage liver disease
NOS: Newcastle‐Ottawa Scale
OR: odds ratio
PICO: population, intervention, comparison, outcome
PRISMA: Preferred Reporting Items for Systematic Reviews and Meta‐Analyses
PROSPERO: International Prospective Register of Systematic Reviews
RAAS: renin‐angiotensin‐aldosterone system
RCT: randomized controlled trial
SGLT2i: sodium‐glucose cotransporter‐2 inhibitors
TIPS: transjugular intrahepatic portosystemic shunt

## Funding

No funding was received for this manuscript.

## Conflicts of Interest

The authors declare no conflicts of interest.

## Supporting Information

Additional supporting information can be found online in the Supporting Information section.

## Supporting information


**Supporting Information 1.** Figure S1: Forest plot of the pooled mean difference in serum creatinine change (mg/dL) with SGLT‐2 inhibitors versus control.

**Supporting Information 2.** Figure S2: Forest plot of the pooled mean difference in eGFR change (mL/min/1.73 m^2^) with SGLT‐2 inhibitors versus control.

**Supporting Information 3.** Figure S3:Forest plot of the pooled mean difference in serum sodium change (mEq/L) with SGLT‐2 inhibitors versus control.

## Data Availability

The data that support the findings of this study are available from the corresponding author upon reasonable request.
