# Supplementary material for: SGLT‐2 Inhibitors for Ascites Management in Liver Cirrhosis: A Systematic Review and Meta‐Analysis of Available Evidence
Source: Int J Hepatol. 2026 Jun 24;2026:7257876. doi: 10.1155/ijh/7257876 (PMC13292109; doi:10.1155/ijh/7257876)

**Supplementary Figure S1:** Forest plot of the pooled mean difference in serum creatinine change (mg/dL) with SGLT-2 inhibitors versus control. The overall pooled MD was −0.05 (95% CI: −0.14 to 0.04; P = 0.30; I² = 69%).


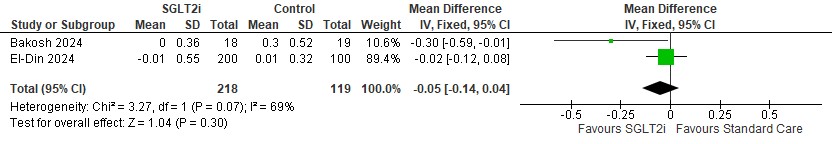


**Supplementary Figure S2:** Forest plot of the pooled mean difference in eGFR change (mL/min/1.73 m²) with SGLT-2 inhibitors versus control. The overall pooled MD was 10.46 (95% CI: −0.70 to 21.62; P = 0.07; I² = 0%).


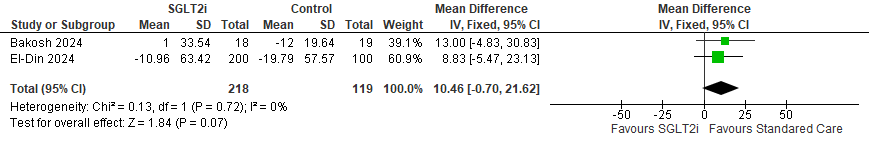


**Supplementary Figure S3:** Forest plot of the pooled mean difference in serum sodium change (mEq/L) with SGLT-2 inhibitors versus control. The overall pooled MD was −0.54 (95% CI: −1.73 to 0.66; P = 0.38; I² = 0%).


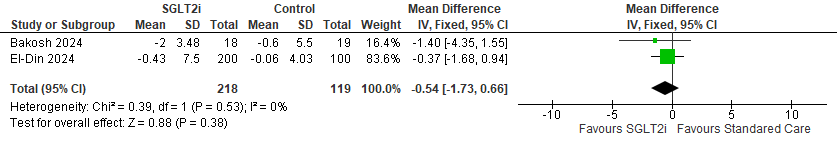

Supplement: Supplementary file 1 — Supporting Information 1. Figure S1: Forest plot of the pooled mean difference in serum creatinine change (mg/dL) with SGLT‐2 inhibitors versus control. [file IJH-2026-7257876-s002.docx]
