# Supplementary material for: SGLT‐2 Inhibitors for Ascites Management in Liver Cirrhosis: A Systematic Review and Meta‐Analysis of Available Evidence
Source: Int J Hepatol. 2026 Jun 24;2026:7257876. doi: 10.1155/ijh/7257876 (PMC13292109; doi:10.1155/ijh/7257876)
Supplement: Supplementary file 2 — Supporting Information 2. Figure S2: Forest plot of the pooled mean difference in eGFR change (mL/min/1.73 m2) with SGLT‐2 inhibitors versus control. [file IJH-2026-7257876-s003.docx]

PRISMA 2020 Checklist

**Manuscript Title:** Sodium-Glucose Cotransporter-2 Inhibitors for Management of Ascites in Liver Cirrhosis: A Systematic Review and Meta-Analysis

**PROSPERO Registration:** CRD420261303781

**Date Completed:** February 14, 2026

# TITLE

| # | Section/Topic | Item | Checklist Item | Location in Manuscript |
| --- | --- | --- | --- | --- |
| 1 | Title | Identification | Identify the report as a systematic review. | Page 1 (Title includes "Systematic Review and Meta-Analysis") |

# ABSTRACT

| # | Section/Topic | Item | Checklist Item | Location in Manuscript |
| --- | --- | --- | --- | --- |
| 2 | Abstract | Structured summary | See the PRISMA 2020 for Abstracts checklist (Background, Methods, Results, Discussion/Conclusions, Registration reported) | Page 2 (Structured abstract with all required elements + PROSPERO registration) |

# INTRODUCTION

| # | Section/Topic | Item | Checklist Item | Location in Manuscript |
| --- | --- | --- | --- | --- |
| 3 | Rationale | Rationale | Describe the rationale for the review in the context of existing knowledge | Pages 3-4 (Introduction section) |
| 4 | Objectives | Objectives | Provide an explicit statement of all objectives or questions the review addresses in terms of PICO | Page 4 (End of Introduction, PICO framework in Methods section 2.3) |

# METHODS

| # | Section/Topic | Item | Checklist Item | Location in Manuscript |
| --- | --- | --- | --- | --- |
| 5 | Eligibility criteria | Eligibility criteria | Specify inclusion and exclusion criteria. Specify the PICO framework used | Pages 5-6 (Section 2.3 - PICO framework with detailed inclusion/exclusion criteria) |
| 6 | Information sources | Information sources | Specify all databases, registers, websites, organizations searched. Specify date when each was last searched | Page 5 (Section 2.2 - PubMed, Scopus, Cochrane Library, Embase, Web of Science up to October 2025) |
| 7 | Search strategy | Search strategy | Present full search strategies for all databases, including any filters and limits used | Page 5 (Section 2.2 - Search strategy with Boolean terms provided; detailed strategies in Supplementary Material) |
| 8 | Selection process | Selection process | State how many reviewers screened each record, whether they worked independently, and how conflicts were resolved | Page 6 (Section 2.4 - Two independent reviewers using Rayyan, disagreements resolved by third reviewer) |
| 9 | Data collection | Data collection process | Describe methods to collect data, how many reviewers, processes for confirming data, how disagreements were resolved | Pages 6-7 (Section 2.5 - Standardized form, pilot-tested, two reviewers independently, author contact attempted) |
| 10 | Data items | Data items (a) | List and define all outcomes for which data were sought | Page 6 (Section 2.5 - Primary: ascites resolution; Secondary: weight, renal function, metabolic parameters, adverse events) |
| 10a | Data items | Data items (b) | List and define all other variables (participant characteristics, intervention details, funding sources) | Page 6 (Section 2.5 - Study characteristics, population, intervention, comparator, duration extracted) |
| 11 | Study risk of bias | Study risk of bias assessment | Specify the tool(s) and version used to assess risk of bias | Page 7 (Section 2.6 - Cochrane RoB 2 for RCTs, Newcastle-Ottawa Scale for non-randomized studies) |
| 12 | Effect measures | Effect measures | Specify the effect measure(s) used in synthesis | Page 7 (Section 2.8 - OR with 95% CI for dichotomous, MD with 95% CI for continuous) |
| 13 | Synthesis methods | Synthesis methods (a) | Describe processes to decide study eligibility, data preparation, methods to synthesize results, explore heterogeneity, sensitivity analyses | Pages 7-8 (Section 2.8 - RevMan 5.4, fixed/random-effects models, I² for heterogeneity, forest plots) |
| 13a | Synthesis methods | Synthesis methods (b) | Describe methods to assess certainty of evidence | Page 7 (Section 2.7 - GRADE approach for main outcomes) |
| 14 | Reporting bias | Reporting bias assessment | Describe methods to assess risk of bias due to missing results (reporting biases) | Page 8 (Section 2.8 - Acknowledged limitation: <10 studies prevents formal assessment) |
| 15 | Certainty assessment | Certainty assessment | Describe methods to assess certainty in the body of evidence | Page 7 (Section 2.7 - GRADE approach with assessment across domains) |

# RESULTS

| # | Section/Topic | Item | Checklist Item | Location in Manuscript |
| --- | --- | --- | --- | --- |
| 16 | Study selection | Study selection (a) | Describe search and selection process, from records identified to studies included, ideally using a flow diagram | Pages 8-9 (Section 3.1) + Figure 1 (PRISMA Flow Diagram) |
| 16a | Study selection | Study selection (b) | Cite studies excluded and explain why | Page 9 (Section 3.1 - Excluded studies with reasons) |
| 17 | Study characteristics | Study characteristics | Cite each included study and present its characteristics | Pages 9-10 (Section 3.2) + Table 1 (Singh 2024, Bakosh 2024, El-Din 2024) |
| 18 | Risk of bias | Risk of bias in studies | Present assessments of risk of bias for each included study | Pages 10-11 (Section 3.3) + Supplementary Materials (RoB 2.0 tables for RCTs, NOS for comparative study) |
| 19 | Results of individual studies | Results of individual studies | For all outcomes and studies, present summary statistics and effect estimates with precision | Pages 11-13 (Sections 3.4-3.6) + Table 2 + Figures 2-4 |
| 20 | Results of syntheses | Results of syntheses (a) | Summarize characteristics and risk of bias among contributing studies | Pages 11-13 (All synthesis sections describe included studies) |
| 20a | Results of syntheses | Results of syntheses (b) | Present results of all statistical syntheses with summary estimates, precision, and heterogeneity measures | Pages 11-13 (OR 2.39, 95% CI 1.47-3.90 for ascites; MD -4.86 kg for weight; I² for all outcomes) |
| 20b | Results of syntheses | Results of syntheses (c) | Present investigations of possible causes of heterogeneity | Pages 11-13 (Clinical heterogeneity discussed: Child-Pugh severity, comparators, drugs) |
| 20c | Results of syntheses | Results of syntheses (d) | Present results of sensitivity analyses | Page 13 (Fixed-effect vs random-effects models compared) |
| 21 | Reporting biases | Reporting biases | Present assessments of risk of bias due to missing results | Page 13 (Acknowledged limitation: too few studies for formal funnel plot assessment) |
| 22 | Certainty of evidence | Certainty of evidence | Present certainty assessments for each outcome | Page 13 + GRADE Table (Supplementary) - GRADE assessments for primary outcomes |

# DISCUSSION

| # | Section/Topic | Item | Checklist Item | Location in Manuscript |
| --- | --- | --- | --- | --- |
| 23 | Discussion | Discussion (a) | Provide general interpretation of results in context of other evidence | Pages 14-16 (Section 4 - Comparison with Dhoop et al., Mantovani et al., and other reviews) |
| 23a | Discussion | Discussion (b) | Discuss limitations of the evidence included | Pages 16-17 (Small number of studies, clinical heterogeneity, mixed designs, short follow-up) |
| 23b | Discussion | Discussion (c) | Discuss limitations of the review processes | Pages 16-17 (Language restrictions, reliance on published data, inability for subgroup analyses) |
| 23c | Discussion | Discussion (d) | Discuss implications for practice, policy, and future research | Page 17 (Clinical implications, need for larger RCTs, longer follow-up studies) |

# OTHER INFORMATION

| # | Section/Topic | Item | Checklist Item | Location in Manuscript |
| --- | --- | --- | --- | --- |
| 24 | Registration | Registration and protocol (a) | Provide registration information or explain if not registered | Page 1 (Title Page) + Page 5 (Section 2.1 - PROSPERO CRD420261303781) |
| 24a | Registration | Registration and protocol (b) | Indicate where protocol can be accessed or explain if not prepared | Page 5 (Section 2.1 - Protocol registered in PROSPERO database) |
| 24b | Registration | Registration and protocol (c) | Describe amendments to registration or protocol | NA - No amendments to protocol |
| 25 | Support | Support | Describe sources of financial/non-financial support and role of funders | Page 1 (Title Page - "This research received no specific grant from any funding agency") |
| 26 | Competing interests | Competing interests | Declare any competing interests of review authors | Page 1 (Title Page - "The authors declare no conflicts of interest") |
| 27 | Availability | Availability of data | Report which materials are publicly available and where | Page 1 (Title Page - "All data included in published article and supplementary files") |

## COMPLIANCE STATEMENT

This systematic review was conducted and reported according to the Preferred Reporting Items for Systematic Reviews and Meta-Analyses (PRISMA) 2020 statement. All 27 items of the PRISMA 2020 checklist have been addressed in this manuscript.

## CHECKLIST COMPLETION

**Checklist Completed By:** Abdallah Al Ghnaimat

**On Behalf of:** The SGLT2i Systematic Review Team

**Date:** February 14, 2026

**Corresponding Author:** Nour Aldin Ahmed (nourmer555@gmail.com)
