# Supplementary material for: SGLT‐2 Inhibitors for Ascites Management in Liver Cirrhosis: A Systematic Review and Meta‐Analysis of Available Evidence
Source: Int J Hepatol. 2026 Jun 24;2026:7257876. doi: 10.1155/ijh/7257876 (PMC13292109; doi:10.1155/ijh/7257876)
Supplement: Supplementary file 3 — Supporting Information 3. Figure S3:Forest plot of the pooled mean difference in serum sodium change (mEq/L) with SGLT‐2 inhibitors versus control. [file IJH-2026-7257876-s001.docx]

**Question:** SGLT2 inhibitors for ascites management

**Setting:** Adults with liver cirrhosis and ascites requiring diuretic therapy

**Bibliography:** Singh et al. 2024 (Dapagliflozin RCT)

Bakosh et al. 2024 (Empagliflozin RCT)

El-Din et al. 2024 (Dapagliflozin comparative study)

| Outcome | № of studies | Study design | Risk of bias | Inconsistency | Indirectness | Imprecision | Other considerations | SGLT2 inhibitors (n) | Control (n) | Relative effect (95% CI) | Absolute effect (95% CI) | Certainty | Importance |
| --- | --- | --- | --- | --- | --- | --- | --- | --- | --- | --- | --- | --- | --- |
| **Change in body weight (kg)** | 2 | randomised trials | serious^a^ | not serious | not serious | not serious | none | 221 | 121 | – | MD **4.86 lower** (7.57 lower to 2.14 lower) | ⨁⨁⨁◯ Moderate^a^ | Critical |
| **Change in serum creatinine (mg/dL)** | 2 | randomised trials | serious^a^ | serious^b^ | not serious | serious^c^ | none | 218 | 119 | – | MD **0.05 lower** (0.14 lower to 0.04 higher) | ⨁◯◯◯ Very low^a,b,c^ | Important |
| **Change in serum sodium (mmol/L)** | 2 | randomised trials | serious^a^ | not serious | not serious | serious^c^ | none | 218 | 119 | – | MD **0.54 lower** (1.73 lower to 0.66 higher) | ⨁⨁◯◯ Low^a,c^ | Important |
| **Change in eGFR (mL/min/1.73 m²)** | 2 | randomised trials | serious^a^ | not serious | not serious | very serious^d^ | none | 218 | 119 | – | MD **10.46 higher** (0.7 lower to 21.62 higher) | ⨁◯◯◯ Very low^a,d^ | Important |
| **Ascites complete response** | 3 | 2 RCTs + 1 prospective | serious^a^ | not serious | not serious | not serious | none | 102/241 (42.3%) | 30/141 (21.3%) | **OR 2.39** (1.47 to 3.90) | **180 more per 1,000** (from 72 more to 300 more) | ⨁⨁⨁◯ Moderate^a^ | Critical |
| **Mortality — RCT subgroup (primary)** | 2 | randomised trials | serious^a^ | not serious | not serious | very serious^d^ | none | 10/41 (24.4%) | 7/41 (17.1%) | **OR 1.60** (0.53 to 4.87) | **76 more per 1,000** (from 78 fewer to 330 more) | ⨁◯◯◯ Very low^a,d^ | Critical |
| **Mortality — overall (3-study pool)** | 3 | 2 RCTs + 1 prospective | very serious^a,e^ | very serious^f^ | not serious | not serious | none | 10/241 (4.1%) | 26/141 (18.4%) | **OR 0.27** (0.13 to 0.56) | **136 fewer per 1,000** (from 105 fewer to 156 fewer) | ⨁◯◯◯ Very low^a,e,f^ | Critical |

**CI:** confidence interval; **MD:** mean difference; **OR:** odds ratio

#### Explanations

1. Downgraded for serious risk of bias: One RCT (Singh et al.) rated as some concerns on RoB 2.0; one non-randomized study (El-Din et al.) included.
2. Downgraded for serious inconsistency: Substantial heterogeneity (I²=69%) with conflicting effects between studies.
3. Downgraded for serious imprecision: 95% CI crosses no effect.
4. Downgraded for very serious imprecision: Very wide 95% CI crosses no effect and includes substantial benefit and harm; small sample size (RCT subgroup: only 17 events across 82 patients).
5. Downgraded for very serious risk of bias: Overall pool dominated (83.9% weight) by a non-randomized prospective trial (El-Din et al.) with insulin (not standard ascites therapy) as comparator and a 100% diabetic population.
6. Downgraded for very serious inconsistency: I²=87%; test for subgroup differences by study design Chi²=10.62, p=0.001, I²=90.6%. The overall pooled estimate reflects between-design heterogeneity and is driven entirely by the non-randomized trial (study-level OR 0.01, 95% CI 0.00 to 0.17). The RCT subgroup, by contrast, showed no significant effect (OR 1.60, I²=0%). The overall pool should not be interpreted as a class-wide mortality benefit.

#### Note on the mortality presentation

Two mortality rows are presented because the test for subgroup differences by study design was highly significant (Chi²=10.62, p=0.001; I²=90.6%), making a single overall estimate misleading. The **RCT subgroup** is the primary mortality result of this review; the **overall pool** is presented for transparency and to document the impact of including the non-randomized study, but is not the primary result and is rated very low certainty for additional reasons of risk of bias and inconsistency.
